# A stochastic model of preventive maintenance strategies for wind turbine gearboxes considering the incomplete maintenance

**DOI:** 10.1038/s41598-024-56436-0

**Published:** 2024-03-08

**Authors:** Hongsheng Su, Yuqi Li, Qian Cao

**Affiliations:** 1 School of Automation and Electrical Engineering, Lanzhou Jiaotong University, Lanzhou, 730070 China; 2 Rail Transit Electrical Automation Engineering Laboratory of Gansu Province, Lanzhou Jiaotong University, Lanzhou, 730070 China

**Keywords:** Electrical and electronic engineering, Pure mathematics, Wind energy

## Abstract

In contemporary large wind farms, the combination of condition-based maintenance (CBM) and time-based maintenance (TBM) has become a prevalent approach in preventive maintenance, which is an indispensable part to ensure the safe, stable and environmental operation of equipment. However, the utilization of an inappropriate maintenance strategy may result in over-maintenance or under-maintenance, leading to unstable equipment operation. Furthermore, the majority of preventive maintenance involves replacement maintenance, which may have adverse effects on the performance of wind turbines with excessive maintenance time. Therefore, this paper takes the gearbox as a case study to introduce the incomplete maintenance parameters into the failure rate function to establish a state model based on the stochastic differential equation (SDE) and describing the state change of incomplete maintenance. And then simulating the state model of the gearbox and the joint preventive maintenance strategy of TBM and CBM through examples, resulting the time-based incomplete maintenance (TBIM) is proposed based on the TBM and the incomplete maintenance, and a new joint preventive maintenance strategy incorporating TBIM and CBM is proposed. Through developing the decision-making process of the maintenance strategy to optimize the inappropriate maintenance which including over-maintenance and under-maintenance and simulating the optimized preventive maintenance strategy to compare with that of TBM and CBM and verify the superiority and effectiveness of the proposed maintenance method.

## Introduction

With the enhancement of global environmental awareness, wind energy, as an environmentally friendly, clean, and sustainable renewable resource, has received widespread attention, and the installed capacity of wind turbines increased by 77.6GW in 2022^1^. However, the complex structures, harsh operating environments, and remote locations of wind turbines create significant challenges for maintenance works, resulting in the wind farms must pay expensive maintenance fees or replace the components inside when a fault occurs^2^. Since the gearbox is one of the components with the highest failure rate in wind turbines, it is necessary to implement preventive maintenance and optimize its maintenance strategies, in order to improve equipment availability, minimize downtime, and reduce maintenance costs^3^.

By inspecting and monitoring the gearbox, symptoms of malfunctions are identified and various maintenance activities are carried out before the malfunction occurs to ensure that the gearbox operates normally and this method is the preventive maintenance strategy which is an effective way to guarantee that the wind turbine is in good condition^4–6^. At present, there are two main types of the preventive maintenance strategy used, namely TBM and CBM. The TBM is implement with fixed time intervals and sufficient maintenance resources are arranged at corresponding implementation time points^7^ and the implementation of CBM is determined by monitoring and diagnosing the state of components, which can predict the development trend of the state and improve the equipment utilization^8,9^. The joint application of TBM and CBM is an advanced preventive maintenance strategy at present and Su establishes the life cycle model of preventive maintenance and discusses the operation behavior and controllability of preventive maintenance of TBM and CBM based on the stochastic process theory^10,11^. Wang establishes an SDE model with Brownian motion as a random term, and uses the SDE model to analyze the current operating state values and reliability of the equipment^12^. Chen compares the SDE model with the ODE model to more accurately reflect the state of the gearbox and analyzes the general operating characteristics of TBM and CBM through the established state model of CBM by introducing a state value function. And after the simulation, a maintenance strategy with TBM as the main part and CBM as the auxiliary is obtained^13^.

However, the preventive maintenance strategy mentioned above, whether it is TBM or CBM, assumes that replacement maintenance is carried out, and it means the equipment immediately recovers as new after the maintenance and the maintenance time is ignored but this is unreasonable in terms of cost and actual operation due to the existence of the downtime^14^. And it is found that when the TBM model is combined with CBM, due to untimely predictions and omissions, making inappropriate maintenance strategy may lead to over-maintenance or under-maintenance^13^. Zhao proposes a state-opportunity maintenance strategy for wind turbines considering incomplete maintenance, in which incomplete maintenance is introduced into the reliability model and combined with state-opportunity maintenance, and the maintenance strategy is formulated by changing the variation of the size of the maintenance factor^4^. Zheng and Wang propose an incomplete maintenance model to improve the preventive maintenance strategy for wind turbines, and an optimal preventive maintenance strategy is obtained by optimizing the incomplete maintenance reliability threshold with the objective function of minimizing the maintenance cost during operation time^15,16^. Therefore, this paper introduces incomplete maintenance to improve the preventive maintenance strategy of wind turbines. At the same time, the maintenance time is considered and modelled and the incomplete maintenance is modeled by introducing the incomplete maintenance parameters which including a decreasing service life factor and an increasing failure rate factor, and they are introduced into the failure rate model in the SDE^4,15,16^. This SDE model can better simulate the state of equipment after non-replaceable maintenance. Through the description of incomplete maintenance, time-based incomplete maintenance (TBIM) has been introduced on the basis of TBM through the strategy of incomplete maintenance, which is more in line with the actual maintenance situation in operation. A joint preventive maintenance strategy of TBIM and CBM proposed in this paper differs from previous approaches that have explored the joint maintenance of TBM and CBM. Specifically, this strategy places greater emphasis on determining whether the state value of equipment has reached the maintenance threshold and analyzing the relationship between the reaching time of the TBIM threshold and the implementing time of CBM within a maintenance cycle to optimize the situation of over-maintenance and under-maintenance. As a result, a preventive maintenance strategy that prioritizes CBM as the primary part and utilizes TBIM as the auxiliary part is established.

The rest of this paper is arranged as follows. Section “The stochastic differential equation (SDE) model” mainly constructs the state transition model of the gearbox and introduce the incomplete maintenance. Section “Parameters’ Calibration” mainly solves the parameters in the SDE. Section “The Example Analysis” directly simulates the gearbox state and reliability through examples, and analyzes the advantages and disadvantages of the joint maintenance of TBM and CBM. And a process diagram of the maintenance strategy within the TBIM cycle is obtained after optimizing the inappropriate maintenance problems including over-maintenance and under-maintenance generated in the simulation, verifying the theoretical feasibility of the model and the preventive maintenance strategy. Based on above, the joint strategy of TBIM and CBM are introduced, and the strategies are verified through simulation.

## The stochastic differential equation (SDE) model

Let the stochastic function *x*(*t*) represent the state of the gearbox. According to the actual operating situation of the gearbox, it is assumed *x*(*t*) = 1 means the gearbox in a brand-new at time *t*, while *x*(*t*) = 0 means it is in a completely breakdown state at time *t*. The state transition model of gearboxes is established based on stochastic differential equation:

1 $${\text{d}}x(t) = - \lambda \left( {x(t),t} \right){\text{d}}t + \mu \left( {x(t),t} \right){\text{d}}B(t)$$

where d*t* and d*B*(*t*) characterize the degradation and the random disturbance of the gearbox; λ (*x*(*t*),*t*) and μ(*x*(*t*),*t*) are the function of the failure rate and the random disturbance coefficient of the gearbox; *B*(*t*) is the Brownian motion.

If there exists a unique solution to Eq. (1) as required in the following text, the following conditions must be satisfied^17^:

### Condition 1

λ* (x*(*t*),*t*) and *μ*(*x*(*t*),*t*) are locally Lipschitz in *x*(*t*) uniformly in *t*, that is, for every *T* and *N*, there is a constant *K* depending only on* T* and *N* such that for all $\left| {x(t)} \right|,\left| {y(t)} \right| \le$ *N* and all $0 \le t \le T$,

2 $$\left| {\lambda \left( {x(t),t} \right) - \lambda \left( {y(t),t} \right)} \right| + \left| {\mu \left( {x(t),t} \right) - \mu \left( {y(t),t} \right)} \right| < K\left| {x(t) - y(t)} \right|.$$

### Condition 2

Coefficients satisfy the linear growth condition:

3 $$\left| {\lambda \left( {x(t),t} \right)} \right| + \left| {\mu \left( {x(t),t} \right)} \right| \le K\left( {1 + \left| {x(t)} \right|} \right).$$

### Condition 3

*X*(0) is independent of $\left( {B(t),0 \le t \le T} \right)$, and $Ex^{2} (0) < \infty$.

Among them, *x*(*t*) and *y*(*t*) are solutions to Eq. (1). For Conditions 1 and 2, scaling the left equation can prove the inequality. Assuming that the random disturbance of the gearbox is independent and stable at any time, and the expected value of the random disturbance is zero, the Condition 3 can be obtained. Satisfying the above three conditions, the SDE Eq. (1) has a unique strong solution.

### The failure rate model

The failure rate of the gearbox in this paper is influenced by three factors. The first is time, meaning that the probability of the gearbox failure inevitably increases over the operating time^18^. The second is the state of the gearbox itself, which with better state values under the same operating time has a lower failure rate. The last is the incompleteness of maintenance, which is introduced especially in this paper. The improvement of the gearbox performance through the incomplete maintenance is usually described by updating the failure rate function^19^. Therefore, the failure rate of a gearbox is abstracted into two parts: one is the basic failure rate affected by time after introducing the incomplete maintenance, and the other is the state impact rate of the basic failure rate change caused by the self-generated state of the gearbox.

#### The incomplete maintenance model

Among various maintenance methods in engineering maintenance, replacement maintenance can restore the state of the equipment to its initial state and is the most ideal maintenance method. In the actual production, however, especially for large equipment such as wind turbines, the cost of replacement maintenance is greater than that of general maintenance, and it shall face prolonged downtime, while the maintenance effect of the minimum maintenance is not significant^20^. Based on the actual operation of wind turbines, the incomplete maintenance with a performance between general maintenance and replacement maintenance is selected. After each instance of maintenance implementation, the state of the wind turbine cannot be restored to a completely new state, but it can be improved greatly, i.e., the equipment state is restored to between "as new" and "as old". This paper introduces service age decline factor *a*_*m*_ and failure rate increasing factor *b*_*m*_ to model the incomplete maintenance^16^. Using the service age decline factor to indicate that the state of the repaired component cannot be restored to a new state, and the initial failure rate of the component after repair can be calculated based on this; The failure rate increasing factor indicates that after the incomplete maintenance, the rate of change in component failure rate shall accelerate with the increase of maintenance frequency. The evolution rules of basic failure rate under incomplete maintenance are as follows:

4 $$h_{m + 1} = b_{m} h_{m} (t + a_{m} T_{m} )$$

where *m* is the cycle of the incomplete maintenance, *m* = 0,1,2…; *h*_*m*_ represents the failure rate function in the m-th cycle of the incomplete maintenance; *T*_*m*_ is the time interval between the m-th and the m + 1-th cycle of the incomplete maintenance; $t \in \left[ {0,T_{m + 1} } \right]$ is the operating time of the wind turbines.

Without considering the incomplete maintenance, the failure rate function of the gearbox follows a three parameters Weibull distribution as it can fit a variety of sample data in reality by adjusting its shape parameters, scale parameters, and position parameters (position parameters is zero)^18^. The expression for the basic failure rate is obtained as follows:

5 $$h_{0} (t) = \frac{\beta }{\eta }\left( {\frac{t}{\eta }} \right)^{\beta - 1}$$

where *β* is the shape parameter and *η* is the scale parameter., *h*_0_(*t*) is the basic failure without incomplete maintenance.

**Figure 1:**
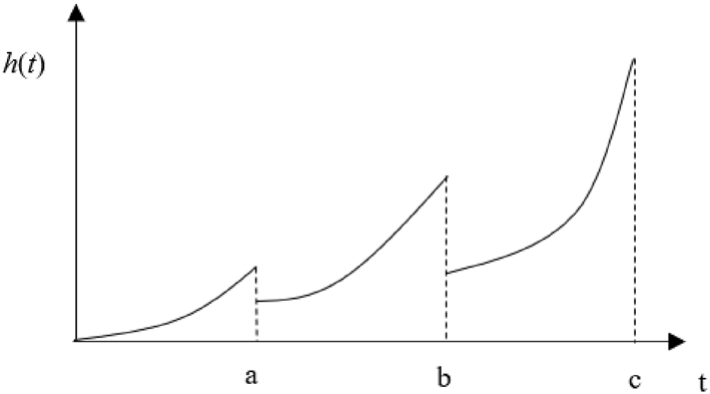
The Evolution process of incomplete maintenance failure rate.

In order to more vividly describe the service age decline factor and failure rate increasing factor on the failure rate function, taking three preventive maintenance cases as an example, the changes in the gearbox failure rate function after each maintenance are shown in Fig. 1.

From Fig. 1, it can be seen that under the combined effect of service age decline factor and failure rate increasing factor, gearbox has a service age regression after each maintenance, resulting in the failure rate function not starting from 0. At the same time, as the number of incomplete maintenance increases, the slope of the failure rate function gradually increases.

#### The state influence rate model

*G*(*x*(*t*)) is the effect of the gearbox state on the failure rate. It is a continuous function with the range of [0,1]. According to Weierstrass’ first approximation theorem^19^ and Taylor expansion^17^, the state influence rate can express as follows:

6 $$g\left( {x(t)} \right) = \frac{1}{1 + \alpha \cdot x(t)}$$

where *α* represents the parameter of the influence of the gearbox state on the failure rate function, also known as regression coefficients.

#### The gearbox failure rate model

Due to the introduction of the incomplete maintenance in this paper, if the polynomial approximation is used, the continuity of the function of gearbox failure rate before and after maintenance should be considered. Through observing incomplete maintenance, the basic failure rate function *h*_*m*+1_ only changes its expression after incomplete maintenance, but it is still continuous. It can be regarded as a piecewise continuous function like Eq. (8), so the whole failure rate function is approximated by polynomials. It can be regarded as a piecewise continuous function, so that the whole failure rate function is approximated by polynomials. The failure rate model of the gearbox after m + 1-th incomplete maintenance is constructed as Eq. (7):

7 $$\lambda_{m + 1} \left( {x(t),t} \right) = h_{m + 1} g\left( {x(t)} \right) = \prod\limits_{i = 0}^{m} {b_{i} } \cdot \frac{\beta }{\eta }\left( {\frac{{t + a_{i} T}}{\eta }} \right)^{\beta - 1} \cdot \frac{1}{1 + \alpha x(t)}$$

8 $$\begin{gathered} h_{2} = b_{1} h_{1} (t + a_{1} T_{1} ) \\ h_{3} = b_{2} h_{2} (t + a_{2} T_{2} ) \\ \cdot \\ \cdot \\ \cdot \\ h_{m + 1} = b_{m} h_{m} (t + a_{m} T_{m} ) \\ \end{gathered}$$

### The random interference model

The interference received by the gearbox is random and independent of the operating time, but its impact on the state of the gearbox, which is called the state fluctuation rate, is related to the gearbox’s current state. Therefore, the recorded state fluctuation rate can express as follows:

9 $$\mu \left( {x(t),t} \right) = k \cdot x(t)$$

where *k* is random interference parameter.

### The maintenance time model

Regarding the maintenance time, this paper adopts a Weibull distribution based Mean Downtime (MDT) modelling estimation method. The MDT is obtained by looking at historical data. a brief description is given below.

Firstly, the maintenance time *T* is obeying a Weibull distribution a with the distribution function:

10 $$D(t) = P(T > t) = e^{ - \tau } ,t > 0$$

where τ = (*ηt*)^*β*^, *β* is the shape parameter and *η* is the scale parameter.

The *T*_MDT_ is

11 $$T_{MDT} = \int\limits_{0}^{\infty } {D(t)dt = \frac{1}{\eta }\Gamma \left( {\frac{1}{\beta } + 1} \right)}$$

where Γ(.) is gamma function.

The variance of downtime is

12 $$T_{{\text{var}}} = \frac{1}{\eta }\left[ {\Gamma \left( {\frac{2}{\beta } + 1} \right) - \Gamma^{2} \left( {\frac{1}{\beta } + 1} \right)} \right]$$

## Parameters’ calibration

After modeling the state equation above, the final gearbox state model is obtained as follows:

13 $${\text{d}}x(t) = - \prod\limits_{i = 0}^{m} {b_{i} } \cdot \frac{\beta }{\eta }\left( {\frac{{t + a_{i} T}}{\eta }} \right)^{\beta - 1} \cdot \frac{1}{1 + \alpha x(t)} + kx(t){\text{d}}B(t)$$

Then the incomplete maintenance parameters in the failure rate model are solved first. Considering the historical failure rate of the wind turbine gearbox and using the fitting method of the failure rate^20^, the following results are obtained:

14 $$a_{m} = \frac{m}{5m + 9}$$

15 $$b_{m} = \frac{13m + 1}{{12m + 1}}$$

Moreover, to solve parameters of the basic failure rate, based on the failure rate function of the gearbox, the reliability function and probability density function of the model are (16), (17):

16 $$R_{m + 1} \left( {x(t),t} \right) = \exp \left[ { - \int\limits_{0}^{t} {\lambda_{m + 1} \left( {x(t),t} \right){\text{d}}t} } \right] = \exp \sum\limits_{i = 0}^{m} {\left[ { - b_{i} \left( {\frac{{t + a_{i} T}}{\eta }} \right)^{\beta } \frac{1}{1 + \alpha \cdot x(t)}} \right]}$$

17 $$f_{m + 1} \left( {x(t),t} \right) = \prod\limits_{i = 0}^{m} {b_{i} } \frac{\beta }{\eta }\left( {\frac{{t + a_{i} T}}{\eta }} \right)^{\beta - 1} \frac{1}{1 + \alpha x(t)} \cdot \exp \sum\limits_{i = 0}^{m} {\left[ { - b_{i} \left( {\frac{{t + a_{i} T}}{\eta }} \right)^{\beta } \frac{1}{1 + \alpha \cdot x(t)}} \right]}$$

The likelihood function *L*(*β*, *η*, *α*) is constructed by Eq. (17), and then taking and simplifying the logarithm of *L*(*β, η*, *α*), the log-likelihood function is obtained:

18 $$\begin{gathered} \ln L\left( {\beta ,\eta ,\alpha } \right) = n\sum\limits_{i = 0}^{m} {\ln \left( {b_{i} \frac{\beta \cdot \alpha }{\eta }} \right)} + \sum\limits_{j = 0}^{n} {\prod\limits_{i = 0}^{m} {\left[ {\left( {\beta - 1} \right)\ln \left( {\frac{{t_{j} + a_{i} T}}{\eta }} \right) - \ln \left( {1 + x(t_{j} )} \right)} \right]} } \\ - \sum\limits_{j = 0}^{n} {\sum\limits_{i = 0}^{m} {\left[ {b_{i} \left( {\frac{{t_{j} + a_{i} T}}{\eta }} \right)^{\beta } \cdot \frac{1}{{1 + \alpha x(t_{j} )}}} \right]} } \\ \end{gathered}$$

In Eq. (18), *n* is the number of data and *x*(*t*_*j*_) is the detection value of the data at time *t*_*j*_.

Then the Newton–Raphson iterative method^21^ is used to solve the parameters of the failure rate model.

In order to obtain the random interference parameter *k*, let Δ*t* be the step size, $0 \le n\Delta t \le T\left( {n = 1,2, \cdot \cdot \cdot ,N} \right)$, and Δ*x*_*n*_(*t)* be the change of the state value. Then we have

19 $$\Delta x_{n} (t) = \int\limits_{0}^{\Delta t} {\lambda \left( {x(t),t} \right){\text{d}}t} + \int\limits_{0}^{\Delta t} \mu \left( {x(t),t} \right){\text{d}}B(t)$$

Substituting *μ*(*x*(*t*),*t*) = *kx*(*t*) into Eq. (19) and transforming it, we have Eq. (20) as follows:

20 $$\int\limits_{0}^{\Delta t} {kx_{n} (t)} {\text{d}}B(t) = \Delta x_{n} (t) - \int\limits_{0}^{\Delta t} {\lambda \left( {x_{n} (t),t} \right){\text{d}}t}$$

The failure rate of the gearbox can be regarded as a fixed value λ_*n*_(*t*) when $\Delta t \to 0$, Plug it into Eq. (20), it can be obtained that:

21 $$k = \frac{{\Delta x_{n} (t) - \lambda_{n} (t) \cdot \Delta t}}{{x_{n} (t) \cdot B_{n} (t)}}$$

Average Eq. (21), we can random the interference parameter *k* as follows:

22 $$k = k_{{{\text{av}}}} = \frac{1}{N}\sum\nolimits_{n = 0}^{N} {\frac{{\Delta x_{n} (t) - \lambda_{n} (t) \cdot \Delta t}}{{x_{n} (t) - B_{n} (t)}}}$$

Estimation of maintenance time parameters by Probability Paper Test and Least Squares with Weibull Distribution^22^:

Taking the logarithm of Eq. (23) :

23 $$\ln ( - \ln (D(t))) = \beta \ln \eta + \beta \ln t$$

Setting γ = ln(− ln(*D*(*t*))), *x* = ln*t*, *A* = β, *B* = *β*ln*η*, Eq. (23) can be rewritten as a linear equation:

24 $$y = Ax + B$$

The parameters *A*, *B* in Eq. (24) are estimated using the Least Squares method, i.e.

25 $$\left\{ {\begin{array}{*{20}l} {\mathop A\limits^{ \wedge } = \frac{{\sum\limits_{i = 1}^{n} {x_{i} y_{i} - n\mathop x\limits^{ - } \mathop y\limits^{ - } } }}{{\sum\limits_{i = 1}^{n} {x^{2}_{i} - n\mathop {x^{2} }\limits^{ - } } }}} \hfill \\ {\mathop B\limits^{ \wedge } = \mathop y\limits^{ - } - \mathop A\limits^{ \wedge } \mathop x\limits^{ - } } \hfill \\ \end{array} } \right.$$

where $\mathop x\limits^{ - } = \frac{1}{n}\sum\limits_{i = 1}^{n} {x_{i} }$; $\mathop y\limits^{ - } = \frac{1}{n}\sum\limits_{i = 1}^{n} {y_{i} }$; *n* is sample size.

## The example analysis

In order to verify whether the model established above can be applied in actual scenarios, part failure data, mean maintenance time and state monitoring values of the gearbox with the same type in a wind farm is shown in Tables 1 and 2 respectively^23^.

**Table 1:** Part failure data of the gearbox.

Number	1	2	3	…	99	100
Life/h	5231	5123	4923	…	5013	5142
Maintenance time/min	697	724	731	…	684	681

**Table 2:** State monitoring values of the gearbox.

Time/h	Amplitude/mm
77	0.352
354	0.371
632	0.471
854	0.499
…	…
5393	7.721
5472	7.987

According to the state monitoring data of the gearbox and the parameter-solving method of the model in Section “Parameters’ Calibration”, it can be obtained that *β* = 2.04, *η*  = 18,258, *α*  = − 0.43, *k* = 0.00127 and $\overline{T}_{MDT}$ = 709 min.

### The state and reliability analysis

Firstly, simulations are performed on the model without considering the incomplete maintenance to analyze the failure rate function and state values of the established model. The failure rate function and gear state function without considering the incomplete maintenance as follows:

26 $$\lambda (x(t),t) = - \left[ {\frac{2.04}{{18258}}\left( \frac{t}{18258} \right)^{1.04} \cdot \frac{1}{1 - 0.43 \cdot x(t)}} \right]$$

27 $${\text{d}}x(t) = - \left[ {\frac{2.04}{{18258}}\left( \frac{t}{18258} \right)^{1.04} \cdot \frac{1}{1 - 0.43 \cdot x(t)}} \right]{\text{d}}t + 0.00127 \cdot x(t){\text{d}}B(t)$$

According to the transformation relation equation of the failure rate function and the reliability function $R(t) = \exp \left( { - \int_{0}^{t} {\lambda (u)du)} } \right)$, we can obtain the reliability function:

28 $$R[x(t),t] = \exp \left[ { - \left( \frac{t}{18258} \right)^{2.04} \cdot \frac{1}{1 - 0.43 \cdot x(t)}} \right]$$

It is required that the gearbox operates normally without any faults occurring when the reliability is greater than 0.9, and needs to maintained when it is less than 0.9.

**Figure 2:**
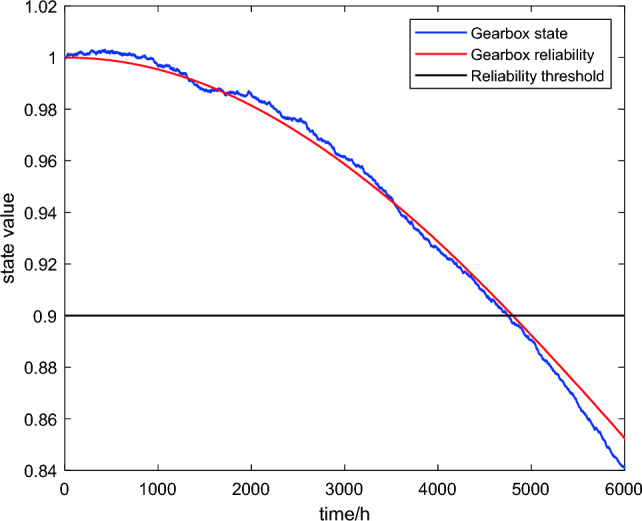
The transition diagram of gearbox’s state and reliability.

Figure 2 shows the process of the state and reliability of the gearbox changing with operating time in SDE.

From the overall trend of the image in Fig. 2, the changing of the transition diagrams of gearbox’s state and reliability is roughly consistent with that of gearbox’s actual state and the state curve and reliability curve of the gearbox basically coincide, both decreasing with time. From the image perspective, firstly, when the reliability of the gearbox reaches the fault threshold of 0.9, the operating time is approximately 4800 h, which is close to the average fault time of 4958 h obtained from Table 1, indicating that the model has certain accuracy. Secondly, by observing the trend of the image, it can be found that the state of the gearbox is not a smooth curve that changes with time since the state is affected by external random disturbances, whereas the overall trend is downward, becoming faster over time. Finally, from Fig. 2, it can be observed that the state and reliability of the gearbox do not show significant changes in the first 1000 h, and their decrease is relatively small in the first 3000 h. It indicates that the maintenance interval of equipment should not be too small, otherwise it is easy to carry out over maintenance and this also provides an idea for proposing incomplete maintenance strategy in the following paper.

In addition, most of the current research on the state model of the gearbox uses ordinary differential equation (ODE), so the ODE is constructed with the Weibull distribution as the failure rate to represent the state transformation model of the gearbox. The state model under ODE is shown in Eq. (29).

29 $${\text{d}}x(t) = - \left[ {\frac{2.73}{{16623}}\left( \frac{t}{16623} \right)^{1.73} \exp \left( {0.24 \cdot x(t)} \right)} \right]{\text{d}}t$$

**Figure 3:**
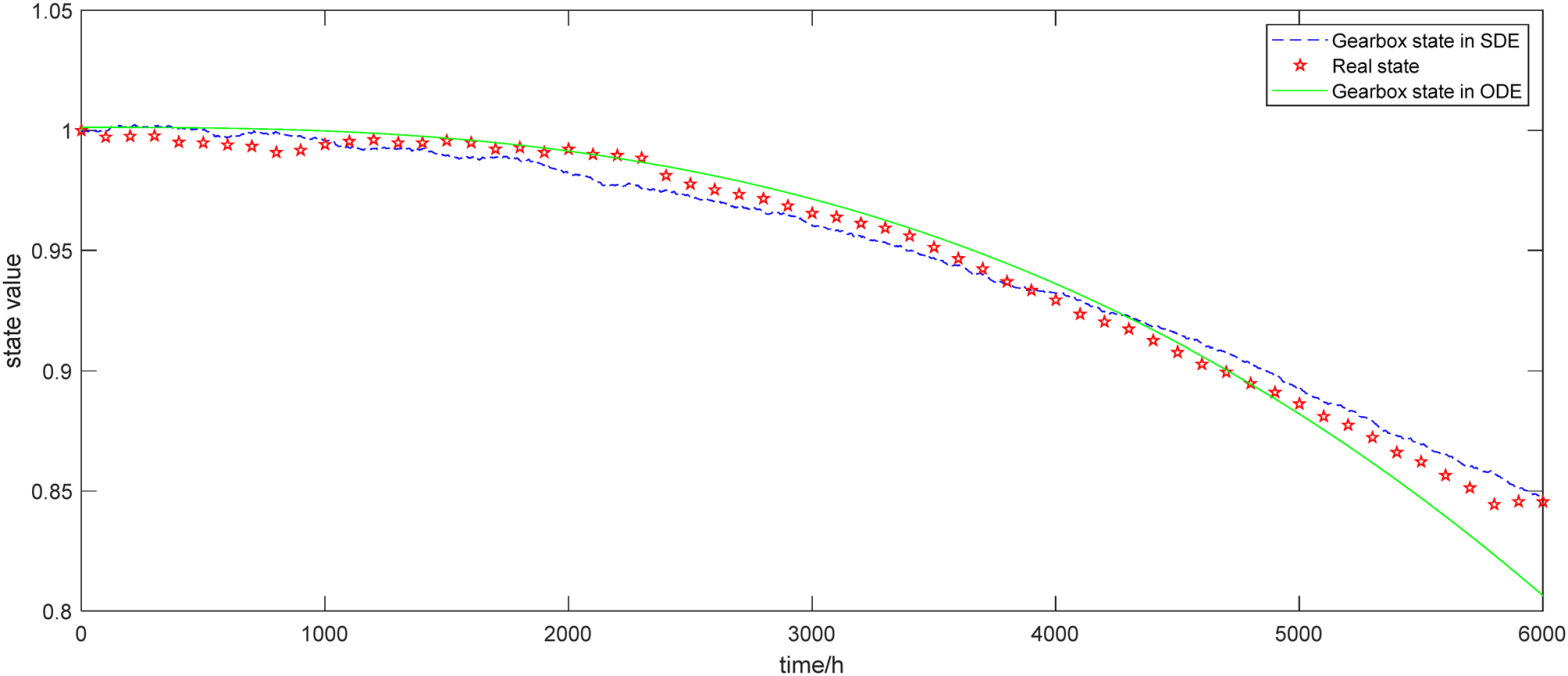
The state of gearbox under different models.

In order to demonstrate that the state values under SDE are closer to the actual gearbox state compared to ODE, a comparison graph of the state values shown in Fig. 3 is obtained by comparing them with the actual state values obtained from the Supervisory Control and Data Acquisition (SCADA) system.

It can be seen from the analysis in Fig. 3, under SDE, the state of the gearbox has a high degree of overlap with the actual state, indicating that the gearbox state model based on SDE is more realistic in describing the state of the equipment. In addition, the state curve under SDE is not smooth, which is caused by the gearbox receiving disturbance from external factors at the same time. Therefore, compared with ODE, SDE is more superior in terms of its overlap with the actual state and its adaptability to the actual production environment.

### The TBM and CBM strategy analysis

Based on the obtained state and reliability transition diagram in Fig. 2, a preventive maintenance strategy for the gearbox is selected. At present, the widely used maintenance strategy in practical production is the joint preventive maintenance strategy of TBM and CBM. The TBM determines whether maintenance is needed by the scheduled maintenance cycle, and the CBM determines that by reaching the fault threshold through the equipment status value.

To begin with a mathematical description of the threshold setting for TBM (or TBIM) and CBM, we need to understand that the essence of CBM is the implementation of maintenance activities guided by information about the state of the gearbox, which implies that there exists a state threshold *X*_*thr*_ such that there is a time *t* = *τ* when^24^:

30 $$x(\tau ) \le X_{thr}$$

Then CBM is required at this time. And the time *τ* at this point can be defined as follows:

31 $$\tau = \inf \left\{ {t > 0;x(t) \le X_{thr} < 1} \right\}$$

The essence of TBM, on the other hand, is the TBM threshold obtained by averaging *τ* with all the information known before the maintenance, then the TBM threshold can be defined as the average of the time at which the CBM threshold is reached for *n* times. The TBM threshold *T*_*TBM*_ is expressed as follows, where *f*_*τ*_ denotes the information known before the maintenance of the gearbox before *τ*_*i*_.

32 $$T_{TBM} = E\left[ {E\left[ {\tau_{i} \left| {f_{\tau } } \right.} \right]} \right] = \frac{1}{n}\sum\limits_{i = 1}^{n} {E\left[ {\tau_{i} \left| {f_{\tau } } \right.} \right]}$$

**Figure 4:**
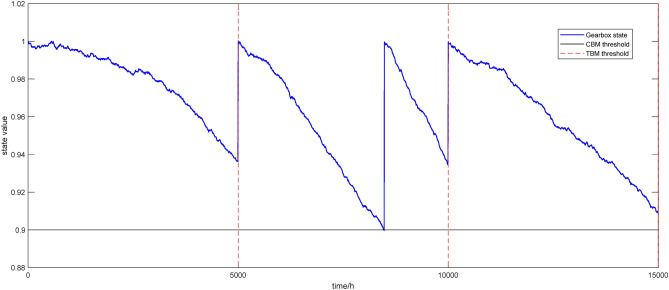
The maintenance strategy of combining TBM and CBM.

In this section, three maintenance cycles are simulated, and the specific maintenance strategy is shown in Fig. 4. According to the reliability analysis of the gearbox in the previous section, we set the CBM threshold *X*_*thr*_ = 0.9, and obtained the TBM threshold *T*_*TBM*_ as 5000 h from the historical monitoring data. In the first TBM cycle, the state value of the gearbox does not fall below 0.9, and thus only the TBM performed at 5000 h. Maintenance time is ignored in the state simulation, and the effect of maintenance time on the strategy is mainly reflected in the cost model. Therefore, the graph represents that the gearbox is immediately restored as new when the maintenance threshold is reached (i.e., the replacement maintenance is performed, but it is obvious that replacement maintenance takes a longer time during the production process, so that the incomplete maintenance will be introduce in the next section). In the second cycle, CBM is implemented on time when the state monitoring value of the gearbox is below 0.9 at 8300 h, which is also the replacement maintenance without considering the maintaining time and restores the equipment to new. And the second TBM is implemented when the TBM threshold is reached at 10,000 h. In the third cycle, CBM should be implemented immediately when the state monitoring value of the gearbox is below 0.9 at 14,500 h. However, on the one hand, if the CBM is implemented at this time, the TBM shall be implemented again in an extremely short period of time due to the next TBM approaching in the near 15,000 h, resulting in the over-maintenance. On the other hand, if CBM is not implemented at this time, the gearbox’s state value shall be lower more than the reliability threshold, and the gearbox is highly likely to malfunction before the next TBM, resulting in the under-maintenance. Thus, although the combination of TBM and CBM can fully leverage their respective advantages and largely overcome their respective shortcomings, there is still room for improvement. Based on the above analysis of the joint preventive maintenance strategy of TBM and CBM, incomplete maintenance is introduced in the next section to further improve the preventive maintenance strategy.

### The incomplete maintenance analysis

**Figure 5:**
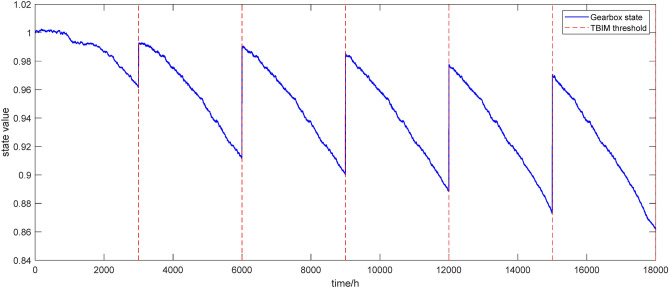
Maintenance strategy of TBIM.

Based on the monitoring values of the gearbox state and reliability and the simulation of traditional TBM and CBM strategies in the first two sections, after analyzing the advantages and disadvantages of the joint preventive maintenance of TBM and CBM, an incomplete maintenance strategy is introduced in this section. The service age decline factor *a*_*m*_ and failure rate increasing factor *b*_*m*_ are introduced into the established state equation^16^. Combining the incomplete maintenance with TBM mentioned in the previous section is called Time Based Incomplete Maintenance (TBIM) in this paper. According to Eqs. (13), (14), and (15), the state model equation based on TBIM is shown in Eq. (33). The state of the gearbox under TBIM is shown in Fig. 5.

33 $$dx(t) = \prod\limits_{i = 0}^{6} {b_{i} } \cdot \frac{ - 2.04}{{18258}}\left( {\frac{{t + 3000a_{i} }}{18258}} \right)^{1.04} \cdot \frac{1}{1 - 0.43x(t)} + 0.00127 \cdot x(t)dB(t)$$

As shown in that Fig. 5, similar to TBM, setting the TBIM maintenance interval, i.e., the TBIM threshold *T* = 3000 h and the incomplete maintenance is implemented *m* = 6 times within 18,000 h of operation. From Fig. 5, the following conclusions can be drawn:

1. Every time the TBIM threshold is reached, the incomplete maintenance shall be performed on the equipment. TBM is the replacement maintenance that is implemented immediately when its time threshold is reached and the maintenance time is considered zero. In contrast to TBM, TBIM is more closely related to production practices, i.e., TBIM is the incomplete maintenance that takes an extremely short and negligible maintenance time to implement when its threshold is reached, and cannot restore the gearbox’s state to a new. For example, the time when TBIM is first reached in the Fig. 5 is 3000 h, and after the incomplete maintenance *x*(3000) < *x*(*T*) < 1.The above indicates that TBIM is closer to actual production scenarios than TBM.
2. Due to the introduction of service age decline factor and failure rate increasing factor in incomplete maintenance, after each instance of the incomplete maintenance for the gearbox, its state cannot be restored to a new, and its failure rate shall increase with the frequency of incomplete maintenance. During a TBIM cycle, as the likelihood of malfunctions increases, it is easy for under-maintenance to occur if only incomplete maintenance is used to prevent malfunctions.
3. During several TBIM cycles, the gearbox should be promptly maintained by combing other preventive maintenance methods, since the characteristics of the incomplete maintenance indicate that the gearbox is more likely to malfunction in the next maintenance cycle after multiple maintenance. For example, it can be seen in the Fig. 5 that in the last two TBIM cycles, the gearbox's state decreases significantly faster and its value has already fallen below the fault threshold of 0.9. In the absence of replacement maintenance, the gearbox’s state becomes increasingly unstable as a result of the rapid increase in the failure rate, although the incomplete maintenance is implemented for the gearbox in the later stage of each operation cycle. So apart from the incomplete maintenance, CBM with replacement maintenance should be added during the maintenance cycle to restore the gearbox's state to the new (i.e., *x*(*t*) restores to 1) and update the failure rate function back to the initial basic failure rate (i.e., *h*(*t*) restores to *h*_0_(*t*)), in order to ensure the operational stability of the gearbox.

Hence, a more reliable preventive maintenance strategy is proposed by combining the TBIM and CBM based on the above conclusions.

### Residual analysis

**Figure 6:**
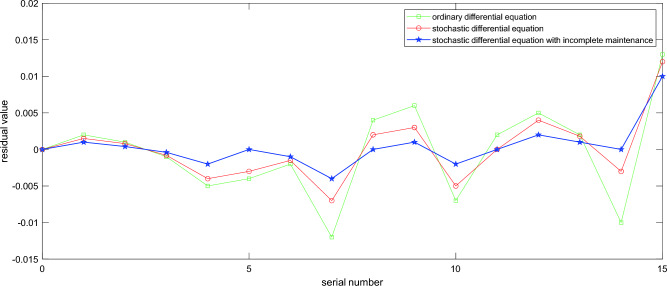
Residual value between the actual state and the expected state of the gearbox in different model.

When the gearbox state models belong to ODE, SDE, and SDE with incomplete maintenance, the residual between the predicted state and the actual state are shown in the Fig. 6.

After calculation, the average residuals of the three maintenance models are 0.0042, 0.0046, 0.0051. Based on the residual trend in the graph, we can draw the following conclusion:

1. The state residual of the equation of state established by ODE has obvious mutation, which cannot well simulate the state change of the gearbox in an emergency. Compared with SDE, the mean residual of ODE is significantly larger, indicating that compared to ODE, SDE can more accurately reflect the state of the device, and has higher applicability and accuracy.
2. The residual and stability of SDE with the incomplete maintenance parameters are clearly due to conventional SDE. This indicates that in the actual operation process of wind turbines, it is difficult to achieve equipment states restoration as new maintenance, while the incomplete maintenance is easier to achieve. Therefore, compared to the actual state, the residual value of SDE with incomplete maintenance is smaller and less prone to sudden changes.

### Maintenance strategies optimization and development

**Figure 7:**
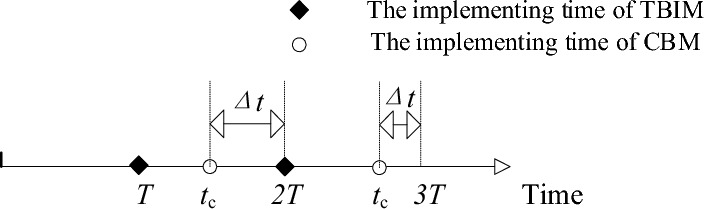
The principle of optimal maintenance strategy for combined application of TBIM and CBM.

In the previous section, through simulating the maintenance strategy of combining TBM and CBM, it is found that over-maintenance and under-maintenance may occur at the TBM threshold reaching time. To avoid them in maintenance strategy of TBIM and CBM, it is possible to establish conditions for the state value and maintenance time after the CBM implementing in order to determine whether the incomplete maintenance is necessary for the current cycle, according to the analysis of the maintenance strategies presented in the previous section. The principle of optimal maintenance strategy for combined application of TBIM and CBM is shown in Fig. 7.

In Fig. 7, *T* is the TBIM maintenance cycle, *t*_c_* i*s the threshold time of CBM, and Δ*t* is the difference between the CBM time and TBIM time within a maintenance cycle.

For CBM, when the state value of the gearbox reaches the CBM threshold during the operation, replacement maintenance shall be implemented, and the gearbox shall be restored as new, i.e., for the entire preventive maintenance strategy, when the CBM threshold is reached, the replacement maintenance has to been implemented to ensure safe operation of gearbox.

For TBIM, when the operating time of the gearbox reaches the TBIM threshold, if CBM is not implemented within the maintenance cycle, the incomplete maintenance will be implemented directly, as a result of which the state value of the gearbox will rebound. And if CBM has been implemented during current maintenance cycle, it is necessary to compare the difference between the implementing time of CBM and TBIM, and observe the state value of the gearbox at this time to determine whether the TBIM is necessary. And if the gearbox malfunctions during operation, it should be immediately shut down for maintenance or replacement of the gearbox.

It is necessary to design an actual maintenance strategy process to avoid over-maintenance and under-maintenance caused by TBIM after the CBM implementing, based on the above analysis and the simulation results of the incomplete maintenance and TBIM in the previous section. Given the uncertainty of gearbox performance, relying solely on the value of Δ*t* at the time threshold of TBIM to determine maintenance implementation is prone to under-maintenance. Thus, it is also necessary to record the state value *x(NT)* at the time threshold of TBIM time and set the state comparison value *x*_*c*_. And if *x(NT)* is greater than *x*_c_ and Δ*t* is lower than Δ*t*_c_, the gearbox will not be maintained and otherwise, the gearbox will undergo the incomplete maintenance at the time threshold and directly enter the next maintenance cycle. As shown in Fig. 7, CBM is implemented during both the first cycle from *T* to 2* T* and the second cycle from 2 to 3* T*, and therefore, it is necessary to compare *x(NT)* and Δ*t*_*c*_ at 2* T* and 3* T*. It can be seen that the TBIM is implemented for the gearbox since Δ*t* at 2* T* is too large or *x*(2* T*) is too small, and TBIM is skipped at 3* T* since Δ*t* at 3* T* is higher than *x*_*c*_.

**Figure 8:**
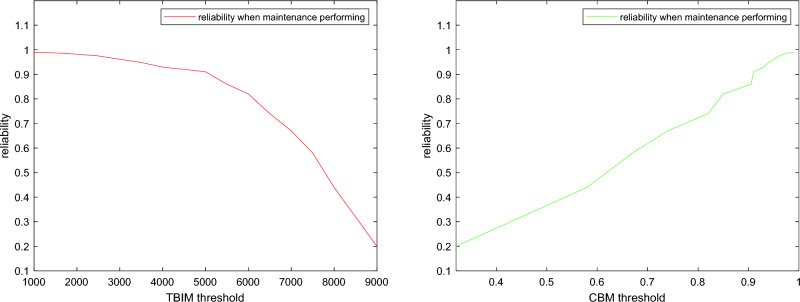
The effect of two maintenance thresholds on the optimization objective.

The effect of the two thresholds on the optimization strategy we show by the reliability before maintenance, from the Fig. 8 it can be observed that when the set CBM threshold is larger, then the reliability of the gearbox before each maintenance is larger. And the smaller the set TBIM threshold is, the greater the reliability of the gearbox before each maintenance, while it can be roughly observed from the Fig. 8 that the reliability at maintenance is 0.9 when the TBIM threshold is chosen to be around 5000 h, and the reliability at maintenance is 0.9 when the CBM threshold is 0.9.

**Figure 9:**
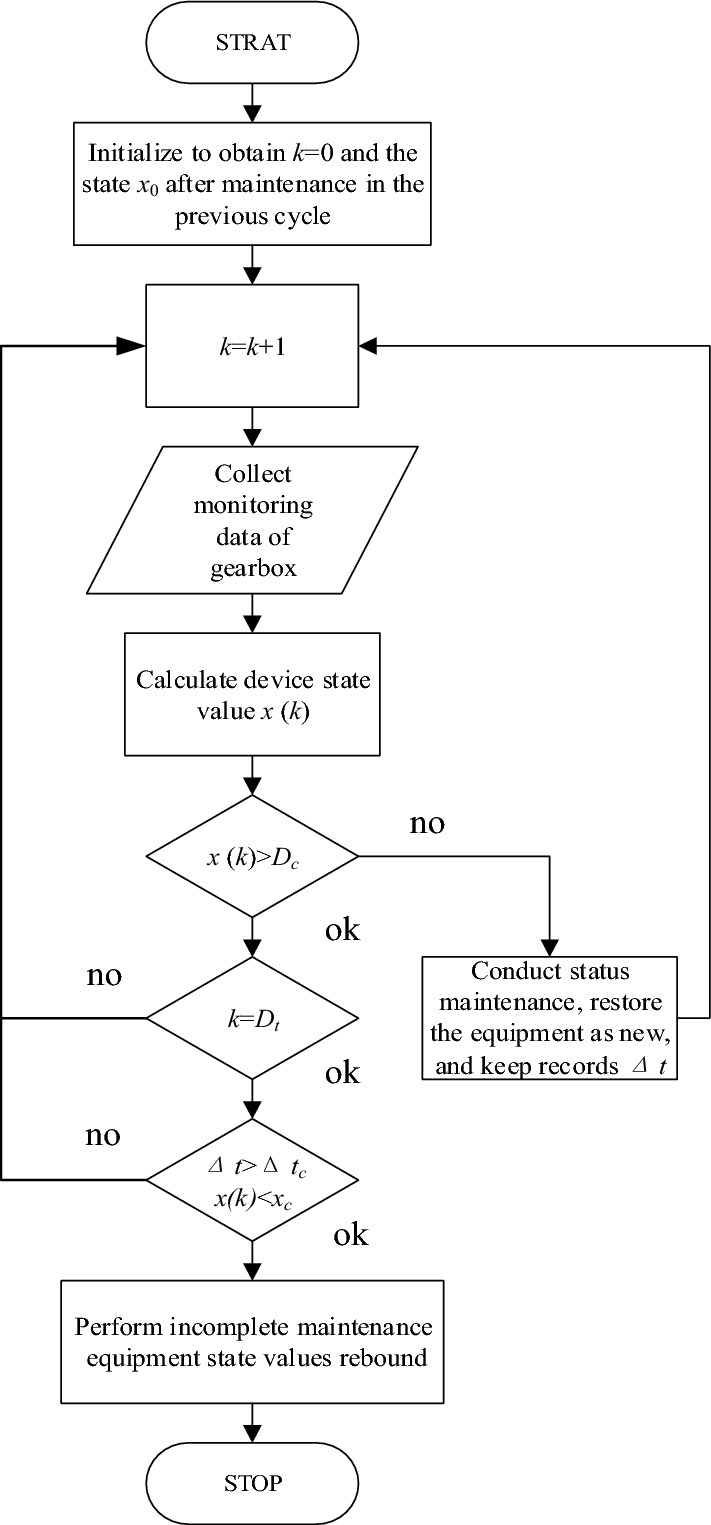
The maintenance decision development.

According to the introduction and optimization of maintenance strategies mentioned above, the maintenance decision-making process based on the joint of TBIM and CBM within a cycle can be obtained, as shown in Fig. 9, where *k* represents time within a maintenance cycle, *D*_*c*_ is the fault threshold of CBM, and *D*_*t*_ is the time threshold of TBIM.

### The joint preventive maintenance of TBIM and CBM analysis

**Figure 10:**
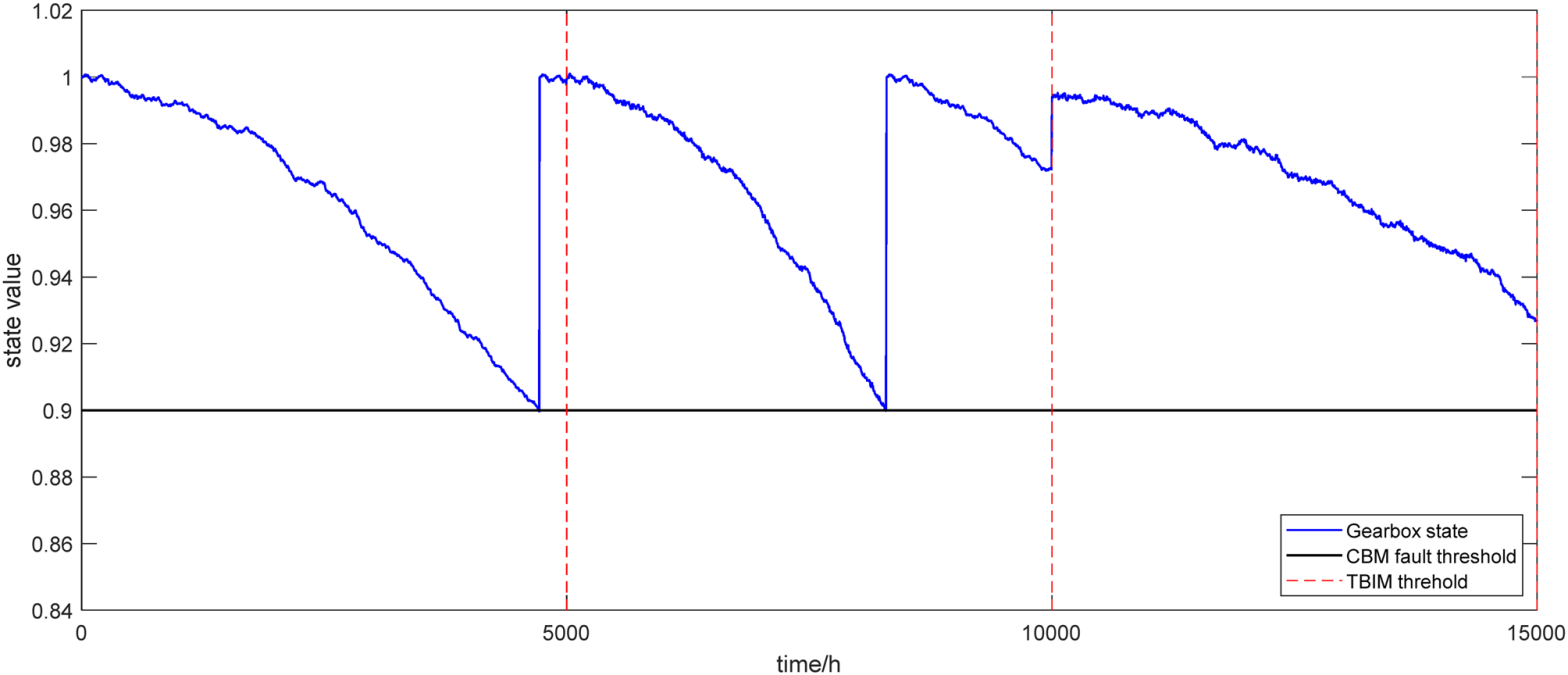
Maintenance strategy of combining TBIM and CBM.

After optimize the maintenance strategies, the frequency of over-maintenance and under-maintenance significantly decreases throughout the entire operation process. Combined with the analyses of the maintenance strategies in Sections “The incomplete maintenance” and “Maintenance strategies optimization and development”, the preventive maintenance strategies based on TBIM and CBM are shown in Fig. 10. This paper sets *D*_*t*_ = 5000 h, *D*_*c*_ = 0.9, Δ*t*_c_ = 500 h and *x*_*c*_ = 0.98. We have maintenance strategy of combining TBIM and CBM within 15,000 h.

It can be found from Fig. 10 that in the first TBIM cycle, the state value of the gearbox is below 0.9 after about 4500 h. At this time, CBM is implemented on time, which is replacement maintenance, resulting that the gearbox is restored as new, the state value is restored to 1, and the fault rate function is updated as before. Approximately 500 h after implementing CBM, the TBIM threshold of 5000 h is reached since replacement maintenance has recently been implemented for the gearbox and its state value has been restored to 1. As shown in Fig. 10, Δ*t* = 450 h is lower than Δ*t*_c_ = 500 h at 5000 h, TBIM will not be performed. In the second TBIM cycle, when the state value of the gearbox is below 0.9 at 8000 h, CBM is implemented on time. After the second replacement maintenance, the gearbox continues to operate and then reaches the second TBIM threshold. In contrast to the first TBIM cycle, after implementing the CBM, the gearbox continues to operate for an additional 2000 h during which its state value significantly decreases, i.e. Δ*t* = 2000 h is higher than Δ*t*_c_ = 500 h and *x*(10,000) = 0.97 is lower than *x*_*c*_ = 0.98 at 10,000 h. Hence, it is necessary to implement incomplete maintenance for the gearbox at this time to restore its state to a certain extent. Starting from 10,000 h of the operation, it is the third maintenance cycle. And during this cycle, the state value of the gearbox does not fall below the CBM threshold, as a result of which only the incomplete maintenance is implemented at the end of this cycle prior to advancing to the next cycle.

From the above analysis, it can be concluded that the combination of TBIM and CBM can greatly leverage their respective advantages and overcome their shortcomings, and can be used to make more reasonable maintenance decisions based on the operating state and time of the gearbox. When compared to the joint maintenance strategy of TBM and CBM, the joint maintenance strategy of TBIM and CBM has the advantage of using CBM to maintain stability and reliability of gearbox operation, thereby preventing failures, and TBIM to ensure that the gearbox state enters the next maintenance cycle with a good value at the end of the current cycle. Additionally, for CBM, it is inevitable to affect production since CBM generally is the replacement maintenance resulting the downtime loss in its actual implementation, while TBIM can minimize the frequency of CBM by keeping the gearbox state as stable as possible. And for TBIM, it is inevitable that the state value of the gearbox decreases faster due to the characteristic of the incomplete maintenance, while CBM can restore the state as new and the failure rate function *h*(*t*) restores to *h*_0_(*t*). In conclusion, TBIM and CBM have their own advantages and disadvantages, but their combination can better ensure the long-term behavior stability of the gearbox.

### Comparison of maintenance costs

Maintenance costs are the main component of wind turbine operating costs, and the formulation of maintenance strategies will affect maintenance costs. We model a maintenance cost as follows:

34 $$C_{total} = \sum\limits_{i = 1}^{N} {\left( {A^{ * } \cdot C_{re} + B^{ * } \cdot C_{in} + C_{mt} } \right)}$$

35 $$C_{mt} = c_{av - mt} \cdot T_{MDT}$$

where *C*_*total*_ denotes the cost of all maintenance, *C*_*re*_ denotes the cost of performing a replacement maintenance, *C*_*in*_ denotes the cost of performing an incomplete maintenance, *C*_*m*_ denotes the cost of damages incurred during maintenance time, *c*_*av*−*mt*_ denotes the average cost of maintenance time damages, and *T*_*MDT*_ is the average maintenance time. A* or B* = 1 indicates that a replacement maintenance or incomplete maintenance is performed, and A* or B* = 0 indicates that a replacement maintenance or incomplete maintenance is not performed. Table 3 shows the various maintenance costs for wind turbines.

**Table 3:** Different maintenance costs for wind turbines.

Type of maintenance costs	*C*_*re*_	*C*_*in*_	*c*_*av*−*mt*_
Cost thousand yuan (CNY)	5.2	2.4	0.8

**Figure 11:**
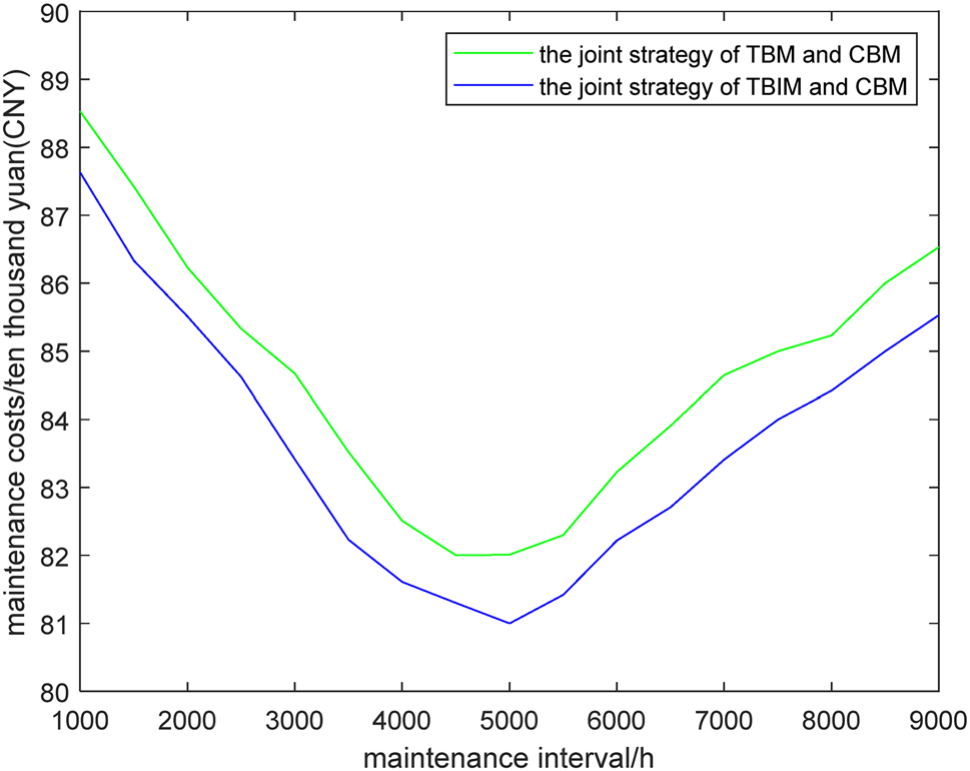
The analysis of maintenance costs for two strategies.

Based on the data in Table 3, Figure 11 shows the impact of setting different maintenance time intervals on gearbox maintenance costs under two preventive maintenance strategies. It can be clearly seen from Fig. 11 that regardless of the maintenance strategy, when the maintenance interval is small or large, the number of maintenances will correspondingly increase or decrease and only by selecting a more appropriate maintenance interval can the maintenance cost be relatively low. It can be clearly observed from Fig. 11 that when the maintenance time interval is about 5000 h, the overall maintenance cost is the lowest. Therefore, setting *D*_*t*_ = 5000 h in the previous section is cost-effective and similar to the TBM threshold based on mathematical derivation as well as optimization. In addition, the maintenance cost of TBIM and CBM joint preventive maintenance is significantly lower than that it of TBM and CBM. This can better save resources and protect the environment in the actual operation of wind turbines.

## Conclusion

In order to optimize the preventive maintenance strategy of its failures, this paper first establishes the SDE model of the gearbox state transformation and in this model, the incomplete maintenance parameters are introduced into the failure rate function, and TBIM is proposed by analyzing incomplete maintenance and combining it with conventional TBM. Then, a joint preventive maintenance strategy of TBIM and CBM is proposed through in-depth analysis of the characteristics of TBIM and the performance rules of gearbox in the environments with random interference. Furthermore, it is comparatively analysis to the differences, advantages and disadvantages of the conventional preventive maintenance strategies of TBM and CBM. Finally, through case simulation analysis, optimization design is carried out for under-maintenance and over-maintenance that are prone to occur in preventive maintenance, and a complete decision-making process of preventive maintenance strategies based on TBIM and CBM is obtained. The preventive maintenance strategies proposed in this paper can not only be applied to wind gearboxes, but also have broad prospects in other preventive maintenance fields.

## Methods

Topical subheadings are allowed. Authors must ensure that their Methods section includes adequate experimental and characterization data necessary for others in the field to reproduce their work.

## Date availability

The data used to support the findings of the study are included within this paper.
